# Ultrasound-guided supraclavicular brachial plexus block as an additive to sevoflurane anesthesia in pediatrics undergoing brachiobasilic arteriovenous fistula operation: randomized controlled clinical trial

**DOI:** 10.1186/s12871-025-03091-1

**Published:** 2025-05-16

**Authors:** Saeid Elsawy, Esraa Fathy, Ahmed Elbadawy, Ashraf Elnaggar, Ahmed Fathy Abdelatif, Hany Elmorabaa, Rasha Hamed

**Affiliations:** 1 Anesthesia and Intensive Care, Faculty of Medicine, Assiut University, Assiut, Egypt; 2 Anesthesia and intensive care, Faculty of Medicine, South Vally University, Qena, Egypt; 3 Vascular surgery Department, Faculty of Medicine, Assiut University, Assiut, Egypt

**Keywords:** Pediatrics patients, Regional anesthesia, End-stage kidney disease, Supraclavicular block

## Abstract

**Background:**

Renal disease is a significant cause of morbidity and mortality in children and is uniquely challenging in the anesthetic management of pediatric patients. Perioperative pain management is a core component in the anesthetic plan. Pediatric regional anesthesia is one of the most valuable and safe tools to treat perioperative pain.

**Methods:**

Sixty pediatric patients with chronic kidney disease scheduled for upper limb superficialization of brachiobasilic arteriovenous fistula to facilitate hemodialysis completed the study. Patients were randomly allocated into two groups; the block group received combined general anesthesia plus ultrasound-guided supraclavicular block, and the control group received general anesthesia only.

**Results:**

The block group recorded a significantly lower fistula maturation duration, more dilatation in basilic vein diameter, lower primary failure rate, postoperative VAS scores, anesthesia consumption, better RASS scores and longer analgesia duration. Moreover, the block group showed more stable hemodynamics with less reduction in MAP than the control group.

**Conclusion:**

Ultrasound-guided supraclavicular brachial plexus block is a safe and feasible adjuvant to general anesthesia that could reduce brachiobasilic AVF maturation time, primary failure rate, control perioperative operative pain and improve the quality of anesthesia recovery by reducing emergence agitation and minimizing sevoflurane anesthesia consumption in pediatric patients with end-stage renal disease.

**Trial registration:**

The study was registered on clinical trial registration (NCT05580094) in October 2022.

## Key message

1. Ultrasound-guided nerve brachial plexus block is a safe and effective anesthetic adjuvant in pediatric anesthesia.

2. It provides robust intraoperative hemodynamic stability in pediatric patients with ESRD.

3. It might play an adjuvant role in enhancing AVF maturation.

## Introduction

Hemodialysis is the predominant therapeutic intervention in managing end-stage renal disease (ESRD). Establishing and maintaining vascular access for hemodialysis in pediatric patients is challenging; therefore, arteriovenous fistula (AVF) is widely used to facilitate hemodialysis vascular access [1]. Surgical intervention for establishing AVF in pediatrics is challenging; it requires an expert surgeon and a long maturation time [2]. However, approximately 60% of newly established arteriovenous fistulas fail to reach adequate maturity for dialysis use, mostly because of inadequate vasodilation and vessel wall remodeling after veno-arterial anastomosis [3]. Successful AVF creation is affected by the preoperative vessel's diameters, arterial inflow, and early postoperative blood flow through the arteriovenous fistulae [4]. Diameter of the vein is the most important limiting factor for primary AVF creation and maturation. Maintaining perioperative adequate blood flow through the fistula is crucial in preventing thrombosis and premature failure of the fistula and promoting its maturation. Although the anesthesia technique could directly affect the venous diameter and blood flow intraoperative and postoperative, no definite evidence exists that any anesthetic technique can influence early patency or long-term outcome of AVF [5]. Regional anesthesia, such as brachial plexus block (BPB), induces sympathectomy that produces vasodilatation, enhancing the blood flow through the upper limb vessels and preventing clamp-induced vasospasm. Regional anesthesia-induced sympathectomy and analgesia start after block and persist several hours postoperatively [4]. We hypothesize that supraclavicular BPB-induced sympathectomy and associated postoperative block could enhance the maturation of brachiobasilic AVF in pediatrics.

## Patients and methods

This is a prospective randomized blind control-blind study. That was conducted in the vascular surgery theatre and post-anesthesia care unit at Assiut University Hospital after obtaining approval from the local ethical committee (IRB 17101925). The study registered on clinical trials on the 6th of October 2022; registration number NCT05580094. Enrollment of patients started on the 14th of November, 2022. 75 patients were enrolled to participate in the study, and 60 patients who completed the study were randomly allocated into 2 groups in a 1:1 allocation ratio:-

- Block Group: 30 patients scheduled for brachiobasilic arteriovenous fistula operation received general anesthesia combined with an ultrasound-guided supraclavicular block by 25% isobaric bupivacaine (3 mg/kg), diluted in saline to a maximum volume of 15 ml.
- Control Group: 30 patients scheduled for brachiobasilic arteriovenous fistula operation received general anesthesia with 15 ml ultrasound-guided supraclavicular saline injection.
- Inclusion criteria: Pediatric patients aged 18 years or younger with ESRD, of both genders, with written consent from the patient's caregiver. Exclusion criteria: Weight < 20 kg, previous ipsilateral attempts at arteriovenous fistula creation, brachial artery diameter less than 1.8 mm or basilic vein diameter less than 2.5 mm at the elbow without a tourniquet, impaired coagulation profile, infection at the anesthetic or surgical site, neurological disorder of the upper limb, ipsilateral subclavian vein stenosis, and refusal of the guardian to sign a consent. Patients were randomly allocated using computer-generated random blocks of 10. Numbers were kept in a sealed opaque envelope; eligible patients were assigned a study number and a sealed envelope opened by the anesthetist immediately before the operation. The study medications were prepared under complete septic conditions in our local research pharmacy and sent to the operating theater in a sealed vial labeled with research medication, patient's name and number. The anesthetist responsible for the block, surgeon, participants (caregivers), and data collectors were all blind to assigned groups.

General anesthesia was induced by fentanyl 0.5 mic/kg, propofol 2 mg/kg slowly infused over 30 s, and cisatracurium 0.15 mg/kg to facilitate ETT intubation after 3 min of 100% O2 preoxygenation. Diameters of the brachial artery and the basilic vein at the elbow were measured by ultrasound preoperative and 10 min after block administration. Anesthesia was maintained with mechanical ventilation (8 ml/kg tidal volume, ventilation rate 12) in 50% O2/air, sevoflurane inhalation (adjusted to keep BIS between 40–60), and muscle relaxant. For preemptive analgesia, intravenous infusion of paracetamol 15 mg/kg thirty minutes before induction of anesthesia. For postoperative nausea and vomiting (PONV) prevention, 4 mg intravenous ondansetron was given 30 min before the end of surgery. Intraoperative hypertension or tachycardia was recognized as a 20% elevation in blood pressure or heart rate from baseline despite adequate anesthesia depth and muscle relaxation, treated with esmolol 100mic/kg increments. Hypotension was treated by administering a bolus of crystalloids and ephedrine increments (12 mg per dose). Bradycardia (< 60 bpm) was treated with 0.01 mg/kg/dose of atropine. At the end of the surgery, neuromuscular blockade was reversed by atropine 0.015 mg/kg and neostigmine 0.04 mg/kg, given after a train-of-four ratio of 0.9. Sevoflurane was turned off, and mechanical ventilation was converted to manual ventilation with 100% O2 at 8 L/min. Extubation was performed only when patients began to breathe spontaneously with a tidal volume ≥ 5 mL/kg, maintained head elevation for 5 s, and obeyed verbal commands with a BIS value of 70. The level of agitation was assessed using the RASS from the closure of inhalational anesthesia up to 30 min after extubation. EA was defined as RASS ≥ 1; dangerous agitation was RASS = 3 or 4 during emergence and in the PACU.

In the PACU and postoperative unit, patients were monitored for EA, the time of the first analgesic request was recorded, and the VAS score. Rescue analgesia was prescribed as intravenous paracetamol 15 mg/kg if VAS < 4, and oxycodone 0.025 mg/kg was prescribed if VAS ≥ 4. Patients were discharged from the PACU based on an Aldrete recovery score ≥ 9.

### Primary outcome measure

Fistula maturation time is assessed by Doppler ultrasonography, starting 4 weeks postoperatively and repeated every 2 weeks until maturity or failure is determined. Mature AVF is defined as a flow volume of more than 600 ml/min measured at 5 cm and 10 cm above the anastomosis, with a vein diameter of > 6 mm and venous depth of < 0.6 mm.

### Secondary outcome measures

Intraoperative hemodynamics was recorded every 15 min till the end of the surgery, anesthetics consumption was recorded every 20 min till the end of the surgery, and emergence agitation was assessed using the RASS from the closure of inhalational anesthesia up to 30 min after extubation. EA was defined as RASS ≥ 1, analgesia duration recorded as the time of the first analgesic request, and postoperative VAS score recorded at PACU, 4 h, 6 h, 12 h and 24 h postoperative.

### Sample size

The sample size was calculated using G*Power analysis based on the results of a previous study [6]; the mean time of fistula maturation at the brachial level was 2.5 ± 0.7. Twenty-seven patients are required in each group to detect a 25% difference in maturation time with α error = 0.05 and 90% power.

### Statistical analysis

Data were analyzed using IBM-SPSS 24.0 (IBM-SPSS Inc., Chicago, IL, USA). Variables normality is assessed using the Kolmogorov–Smirnov test. Chi-square/Fisher's exact/Monte Carlo exact test assessed the frequencies among different groups. Parametric data was analyzed by an independent sample t-test. Nonparametric data was analyzed by Mann–Whitney test (Boezaart score, RASS score). Data expressed as numbers, percentages, and mean ± SD. Repeated-measure analysis with Bonferroni correction was used to analyze repeated measures (VAS, MAP, and HR). A *P*-value < 0.05 was considered statistically significant.

## Results

**Fig. 1:**
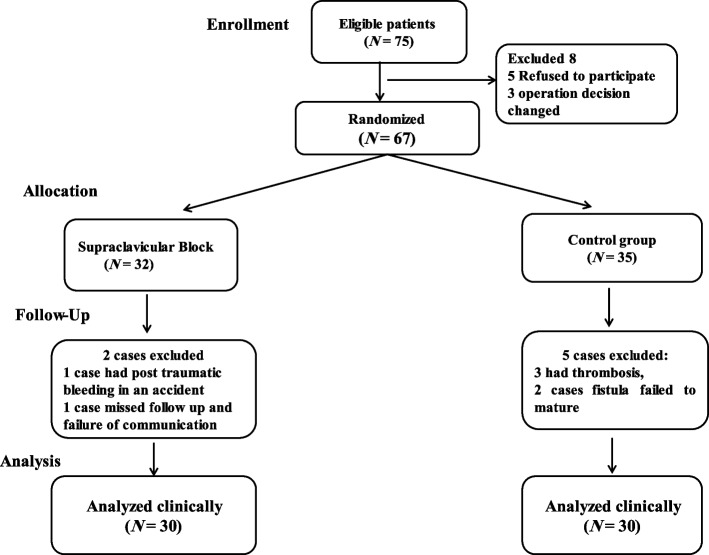
Flow chart

Sixty out of seventy-five patients completed the study, and 8 patients were excluded before randomization (Fig. 1). Sixty-seven patients were randomized into 32 patients in the block group and 35 in the control group. Seven patients were excluded in the follow-up postoperative visits; no cases in the block group were excluded due to failure, thrombosis, or required any additional intervention to assist maturity, while in the control group, 3 cases were excluded due to thrombosis and required thrombectomy to regain flow and 2 cases fistula failed to mature. One case in the block group was excluded due to traumatic fistula rupture, and another case also in the control group was excluded due to loss of follow-up visits and failure of communication (Fig. 1).

**Fig. 2:**
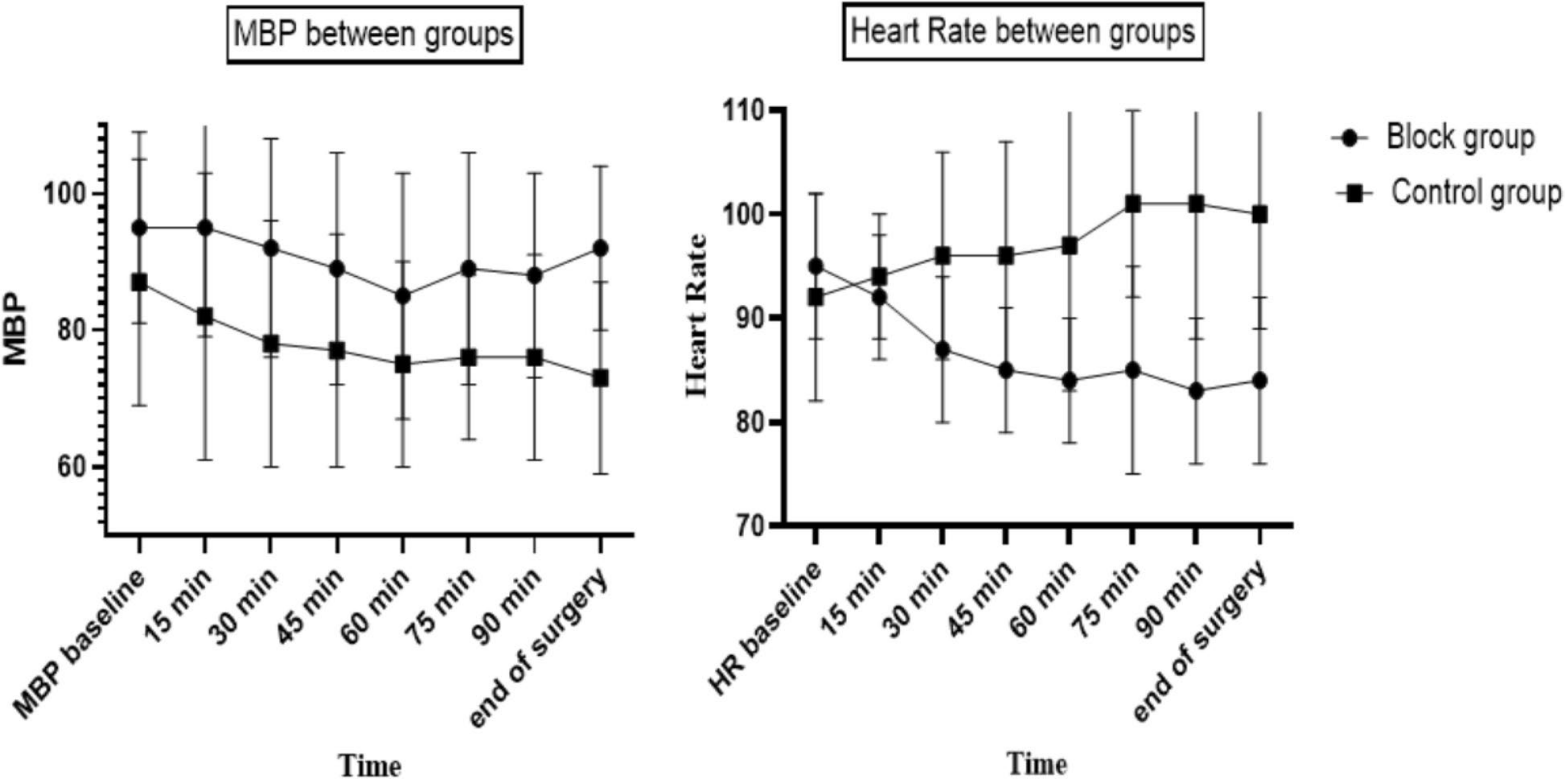
Hemodynamics between study groups

**Fig. 3:**
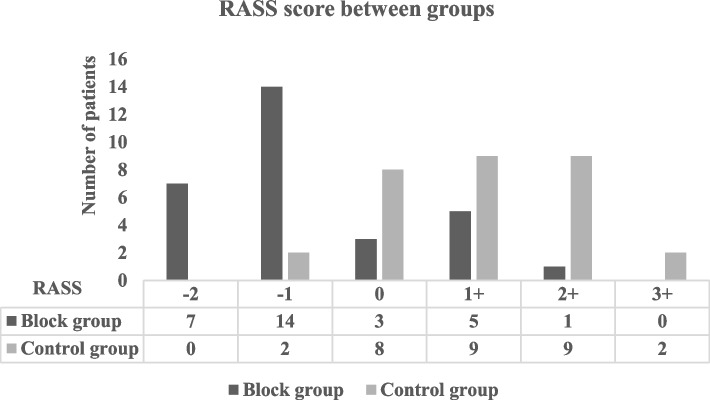
Emergence agitation between study groups

**Fig. 4:**
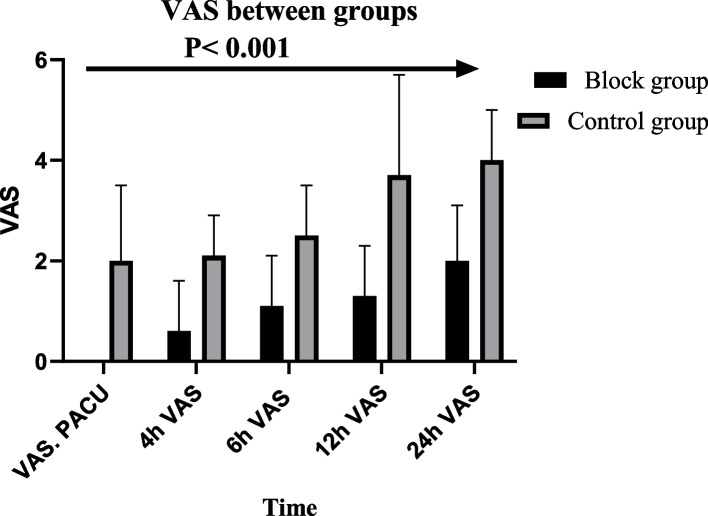
VAS scores between groups

No statistical difference between the study groups was found in demographic data or the baseline hemodynamics. However, a significant difference was found in hemodynamics after supraclavicular block, with robust maintenance of hemodynamics among the block group, less reduction in MAP (*P* value < 0.001) and lower heart rate (Fig. 2). A significant increase in the basilic vein diameter after the block was noticed in the supraclavicular block group than the control group, with a mean diameter difference of 1 ± 0.3 in the block group versus 0.3 ± 0.1 in the control group, *P* < 0.001 (Table 1). The time needed for the brachiobasilic fistula to reach maturity was significantly lower in the block group than the control group, 12 ± 1.7 VS. 15 ± 1.6 weeks, *P* < 0.001 in both groups, respectively (Table 1). Intraoperative anesthesia MAC was significantly lower in block group 2 ± 0.3 compared to 2.5 ± 0.3 in the control group, *P* < 0.001 (Table 1). Anesthesia emergence quality was better in the block group, where the incidence of emergence agitation in the block group was 20% versus 66% in the control group with *P* < 0.001 (Table 1) and (Fig. 3). Pain scores in the first 24 h postoperative were significantly lower in the block group compared to the control group (Fig. 4).

**Table 1:** Demographic and perioperative operative data and fistula maturation time

	**Block Group** ***N* = 30**	**Control Group** ***N* = 30**	***P*-value**
Age	11 ± 2	10 ± 1.5	0.1
Range	(7–15)	(8–13)	
Sex			
Male	13	15	
Female	17	15	0.3
BMI	19 ± 3	18 ± 2.5	0.3
Mean MAC consumption	2 ± 0.3	2.5 ± 0.3	< 0.001
Incidence of EA	6 (20%)	20 (66%)	< 0.001
First analgesic request (h)	6.4 ± 1	1 ± 0.5	< 0.001
Mean difference in basilic vein diameter (cm)	1 ± 0.3	0.3 ± 0.1	< 0.001
Fistula maturation time (weeks)	12 ± 1.5	15 ± 1.6	< 0.001

## Discussion

This study indicated that supraclavicular BPB could reduce the duration of brachiobasilic AVF maturation time, primary failure rate, emergence agitation and postoperative pain scores compared to general anesthesia. To the best of our research, this is the first randomized trial that focused on the effect of regional anesthesia on brachiobasilic AVF maturation time in the pediatric population.

AVF is the access of choice for maintaining hemodialysis. However, the procedure has a high early failure rate, mostly due to surgery-induced sympathetic overflow that causes spasm of the radial artery [7]. Regional anesthesia is considered the optimal anesthetic modality for AVF creation, as the venous dilation from sympathetic blockade improves fistula creation and maturation [8]. Moreover, regional anesthesia maintains more stable hemodynamics than general anesthesia. The introduction of real-time ultrasound guidance increased the success rate and reduced the complications of regional anesthesia [7]. In pediatrics, peripheral nerve blocks are an emerging effective and safe technique for postoperative analgesia. PNBs provide efficient, site-specific analgesia and reduce opioid consumption and opioid-induced side effects. Furthermore, sympathectomy-induced vasodilatation is of great importance in AVF creation and maturation [9]. In situations where the veins were first believed to be too small, as in pediatrics, RA was found to successfully change the operative decision from graft to autogenous conduit and proximal to more distant vein. This correlates with postoperative enhancement of arterial and AVF blood flow [8]. Supraclavicular brachial plexus block has an anatomical advantage as the block is done at the level of the brachial plexus trunks, where the brachial plexus components are grouped tightly; this facilitates a single-point injection and is believed to provide very rapid onset [9]. Arterial and venous vasodilatation that follows BPB could minimize the technical difficulties of the anastomosis, particularly in small vessels as in pediatrics. Moreover, BPB-induced vasodilatation encourages blood flow through the created AVF and reduces early failure by improving the arterial inflow, increasing venous compliance, and reducing the pulsatility index [10, 11].

In accordance with our results, Terkanlioglu et al. compared the effect of brachial plexus block and local anesthesia as anesthetic techniques in AVF operation; they found that brachial plexus block provides more favorable conditions for AVF creation through block-induced vasodilation, which enhances fistula blood flow [12]. Cole et al. found that regional anesthesia for AVF surgery is associated with a lower risk of reoperation and shorter postoperative hospital stays [13]. Furthermore, Aitken et al., in their randomized trial, compared BPB mainly supraclavicular approach with regional anesthesia for radiocephalic and brachiocephalic AVF creation. They found that BPB improved three-month patency rates, promoted fistula maturation and reduced primary thrombosis rates. They attributed these findings to the vasodilatory effect of BPB that ensures more laminar blood flow to the created AVF; this effect starts immediately after creation and extends up to a few days postoperative [10]. Lee et al. retrospectively analyzed the plausibility of RA as an anesthesia modality in AVF revision interventions. RA, in comparison to local anesthesia, is found to be more effective and safer, as local anesthesia usually requires repeated injections; this causes pain and harbors the potential danger of an inadvertent intravascular injection, increasing the risk of local anesthetic toxicity. Moreover, if the surgical field needs to be extended, it won't be easy to cover the whole required segment. In comparison with general anesthesia, RA provided adequate perioperative analgesia and lower cardiorespiratory complications in patients with ESRD. As AVF management is an integral part of dialysis patients, attention to the anxiety, discomfort, and pain these patients experience is required [14].

In contrast with the results of the present study, we found one study where the authors found no advantage of regional over general anesthesia in the early outcome of AVF. A possible explanation of their contradicting results is their choice of adult population with a preoperative large arterial and venous diameter, where the preoperative arterial diameter was more than 2 mm, and the preoperative venous diameter was more than 2.5 mm; this probably masked the vasodilatory effect induced by BPB [15]. The present study is limited by its relatively small sample size and lack of midterm and long-term outcomes of BPB effects on AVF. Also, injecting saline in the control group in pediatrics, despite approved from our local ethical committee, may not be universally accepted.

## Conclusion

Ultrasound-guided supraclavicular brachial plexus block is a safe and feasible adjuvant to general anesthesia that could reduce brachiobasilic AVF maturation time, primary failure rate, control perioperative operative pain and improve the quality of anesthesia recovery by reducing emergence agitation and minimizing sevoflurane anesthesia consumption in pediatric patients with end-stage renal disease.

## Data Availability

No datasets were generated or analysed during the current study.
